# The role of pelvic coronal parameters in determining sagittal balance: a prospective radiographic analysis of pelvic spinopelvic alignment in Chinese asymptomatic Han adolescents

**DOI:** 10.3389/fped.2025.1565338

**Published:** 2025-04-14

**Authors:** Hao Qi, ZengHui Zhao, Feiyu Zu, Chenchen Wang, Chenxi Wang, Zuzhuo Zhang, Siyuan Wang, Shuo Yang, Haiyun Niu, Rui Xue, Zhiyong Hou, Wei Chen, Di Zhang

**Affiliations:** ^1^ Department of Spine Surgery, Hebei Medical University Third Hospital, Shijiazhuang City, Hebei, China; ^2^ Department of Radiology, Hebei Medical University Third Hospital, Shijiazhuang City, Hebei, China; ^3^ Department of Joint Surgery, Hebei Medical University Third Hospital, Shijiazhuang city, Hebei, China; ^4^ Key Laboratory of Biomechanics of Hebei Province, Shijiazhuang City, China; ^5^ Department of Orthopaedic Surgery, Hebei Medical University Third Hospital, Shijiazhuang City, Hebei, China; ^6^ Department of Orthopaedic Surgery, Tiemenguan People's Hospital, Tiemenguan City, Xinjiang, China

**Keywords:** spinopelvic balance, pelvic tilt, pelvic sacral angle, iliac tilt, sacral-femoral-pubic angle, Pubo-Hilgenreiner Distance

## Abstract

**Background:**

Adolescent idiopathic scoliosis (AIS) is a complex three-dimensional spinal deformity where sagittal alignment significantly influences clinical outcomes. This study aims to analyze spinopelvic balance parameters in asymptomatic adolescents without scoliosis, examining the correlation between pelvic coronal parameters and sagittal balance parameters to validate their use as preliminary indicators in assessing pelvic rotation.

**Methods:**

A prospective study was conducted involving 354 adolescents (199 females, 155 males) aged 6–18 years who underwent full-length anteroposterior (AP) and lateral spine radiographs at The Third Hospital of Hebei Medical University between October 2022 and December 2023. Ethical approval and informed consent were obtained. Radiographic assessments included measuring pelvic incidence (PI), pelvic tilt (PT), sacral slope (SS), pelvic sacral angle (PSA), and other related parameters. Statistical analysis was performed using SPSS software.

**Results:**

Significant gender differences were observed in Risser sign distribution and pelvic tilt, with females exhibiting more advanced skeletal maturity. Positive correlations were found between pelvic incidence and pelvic tilt (r = 0.41), and pelvic incidence and sacral slope (r = 0.57), while negative correlations were noted between pelvic incidence and pelvic sacral angle (r = −0.43). Strong correlations between sagittal parameters and newly introduced coronal parameters, such as sacral-femoral-pubic angle and Pubo-Hilgenreiner Distance, were also identified.

**Conclusion:**

The study demonstrates that parameters like Iliac tilt, Pelvic sacral angle, Sacral-femoral-pubic angle, and Pubo-Hilgenreiner Distance correlate well with traditional spinopelvic parameters and can be used to assess pelvic sagittal balance in clinical settings. Future research should focus on longitudinal studies to further validate these findings.

## Introduction

Adolescent idiopathic scoliosis (AIS) is a structural, lateral, and rotational curvature of the spine that affects 1%–3% of children, typically arising around puberty. AIS is a well-documented 3D spinal deformity where sagittal alignment plays a crucial role in clinical outcomes and should not be overlooked in the evaluation and management of the condition (1, 2). The assessment of spinal deformity must include pelvic parameters to ensure a comprehensive evaluation and accurate diagnosis (3). Duval-Beaupere et al. (4) initially described three classic pelvic angles in spinal surgery: pelvic incidence (PI), pelvic tilt (PT), and sacral slope (SS). These angles were evaluated in our previous study, which revealed unique characteristics in the pelvic and sagittal parameters of healthy adolescents. However, we only assessed the traditional pelvic-spine parameters (5).

PT is a recognized positional measure associated with sagittal plane spinal deformities. According to current knowledge of sagittal alignment, some researchers have suggested that the misalignment of the femoral heads can affect measurement accuracy (6, 7). Additionally, the difficulty in accurately identifying the midpoint of the upper endplate of S1 can lead to inaccuracies in measuring PT (8). Therefore, it is necessary to incorporate other parameters that reflect sagittal spinal balance in clinical practice to ensure more accurate assessments, such as the Pelvic Sacral Angle (PSA) and the Iliac Tilt (IT). In the study by Doi T (9), the concept of the iliac cortical density line was introduced, which is also the line we observed when measuring pelvic-spine sagittal parameters. This line can be seen as a straight line several centimeters in length along the anterior border of S1. IT, formed by the iliac cortical density line and a vertical line, is considered to reflect PT. The Pelvic Sacral Angle (PSA) is defined as the angle between the line drawn from the anterior point of the sacral endplate to the superior margin of the pubic symphysis and the horizontal line (10). This angle was introduced to evaluate sagittal balance within the pelvis. Siebenrock (11) utilized Pelvic Inclination to assess the inclination of the pelvis. To avoid abbreviation conflicts with pelvic incidence, we used the term PSA. The application of these angles in evaluating sagittal spinal balance in children has been scarcely explored in research.

In recent years, researchers have proposed and acknowledged the sacro-femoral-pubic (SFP) angle as a parameter for estimating PT from the coronal view (12–14). However, a study by Ghandhari et al. (15) examined the correlation between PT and the SFP angle, concluding that the SFP angle exhibited a weak correlation with PT. This finding suggests that the SFP angle is not a reliable proxy for PT measurements in both scoliotic and healthy populations. Another parameter for assessing sagittal plane rotation of the spine from a coronal view in this study is the distance between the upper border of the pubic symphysis and the line connecting the outer edges of the bilateral acetabula. This measurement method was introduced in Yang Y's study to evaluate pelvic rotation (16). Although these two indicators serve as preliminary assessments and the accurate evaluation of PT requires sagittal plane parameters, we consider them to be convenient preliminary indicators for assessing pelvic sagittal tilt in clinical settings when precise angle measurements are needed on coronal radiographs.

We hypothesize that coronal pelvic parameters can serve as preliminary indicators for sagittal balance in adolescents with AIS. This study aims to analyze the correlation between pelvic coronal parameters and sagittal balance parameters to validate the use of coronal measurements as preliminary or supplementary indicators in clinical settings.

## Methods

### Study design

This is a prospective study conducted through a radiographic review of 354 Chinese asymptomatic Han adolescents (199 females and 155 males) who underwent full-length anteroposterior (AP) and lateral whole-spine radiographs at The Third Hospital of Hebei Medical University between October 2022 and December 2023. No additional radiographs were obtained.

This study was conducted in accordance with the Declaration of Helsinki. Ethical approval was obtained from the Ethics Committee of The Third Hospital of Hebei Medical University (Approval No. K2022-067-1) on July 15, 2022. Data for this study were accessed and collected between October 1, 2022, and December 31, 2023. All participants and their guardians were fully informed about the study objectives and procedures before their enrollment, and signed informed consent specifically for this research project.

Patients exhibiting any signs of scoliosis on radiographic examination were excluded from the study, ensuring that all participants were categorized as “asymptomatic adolescents.” Imaging data that presented unclear anatomical structures or rotational deformities were also excluded. Additionally, radiographs showing bilateral inconsistency in the Risser sign were also excluded. Patients with a history of spinal trauma, infection, or surgery, were not included in the analysis. For each patient, a comprehensive visualization of all spinal and pelvic landmarks, including the pelvis and femoral heads, was obtained from both coronal and sagittal x-rays.

### Radiographic assessment

**Figure 1:**
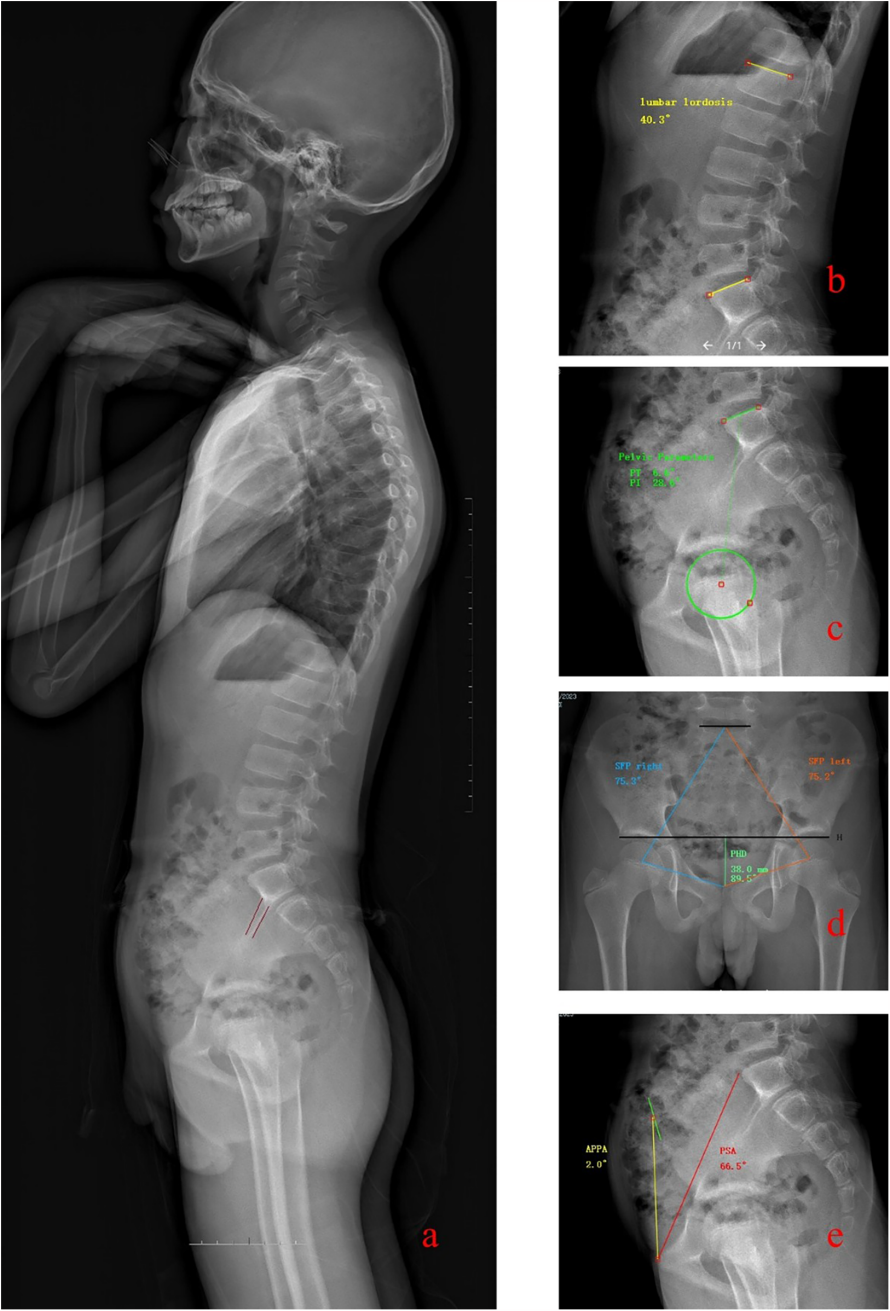
Figure illustrates the methods used for measuring various spinal and pelvic parameters: Panel a: Depicts the measurement method for the Iliac Tilt (IT). This involves identifying the two density lines on the iliac cortex, and the angle formed with a vertical line is recorded. Panel b: Demonstrates the measurement of Lumbar Lordosis (LL), which is the angle between the superior endplates of the L1 and S1 vertebrae. Panel c: Shows the techniques for measuring Pelvic Incidence (PI), Pelvic Tilt (PT), and Sacral Slope (SS). These measurements provide insights into the spatial orientation and inclination of the pelvis. Panel d: Details the measurements of the Sacral-Femoral-Pubic angles (SFP) on both left and right sides and the Pubo-Hilgenreiner Distance (PHD), crucial for assessing coronal pelvic parameters. Panel e: Explains the measurement of the Pelvic Sacral Angle (PSA) and the Anterior Pelvis Plane Angle (APPA), which describe the angular relationships of the sacral and anterior pelvic planes relative to the horizontal and vertical axes, respectively.

At our hospital, standardized radiographic protocol requires obtaining full-length x-rays of the entire spine with patients standing in a natural, upright posture. The imaging extended from the head to the proximal femora. The detailed measurement methods and grading systems are as follows (The schematic diagram of the measurement is shown in Figure 1):

On the coronal view, the Risser sign grading for each subject was first recorded.

Sacral-femoral-pubic angle (SFP) is formed by drawing lines between the midpoint of the upper sacral endplate, the centroid of one acetabulum, and the upper midpoint of the pubic symphysis. Record the angles for the left and right sides separately as SFP left (SFPl) and SFP right (SFPr).

Pubo-Hilgenreiner Distance (PHD) is defined as the distance between the upper border of the pubic symphysis and the line connecting the outer edges of the bilateral acetabula.

### On the lateral view

Pelvic incidence (PI) is defined as the angle between a line perpendicular to the midpoint of the sacral plate and a line extending to the axis of the femoral heads.

Pelvic tilt (PT) is the angle between the vertical line and a line from the midpoint of the sacral plate to the axis of the femoral heads, reflecting the orientation of the pelvis.

Sacral slope (SS) is the angle between the horizontal line and the sacral plate, indicating the inclination of the sacral endplate.

Lumbar lordosis (LL) is measured as the angle formed between the superior endplates of the L1 vertebra and the S1 vertebra.

**Figure 2:**
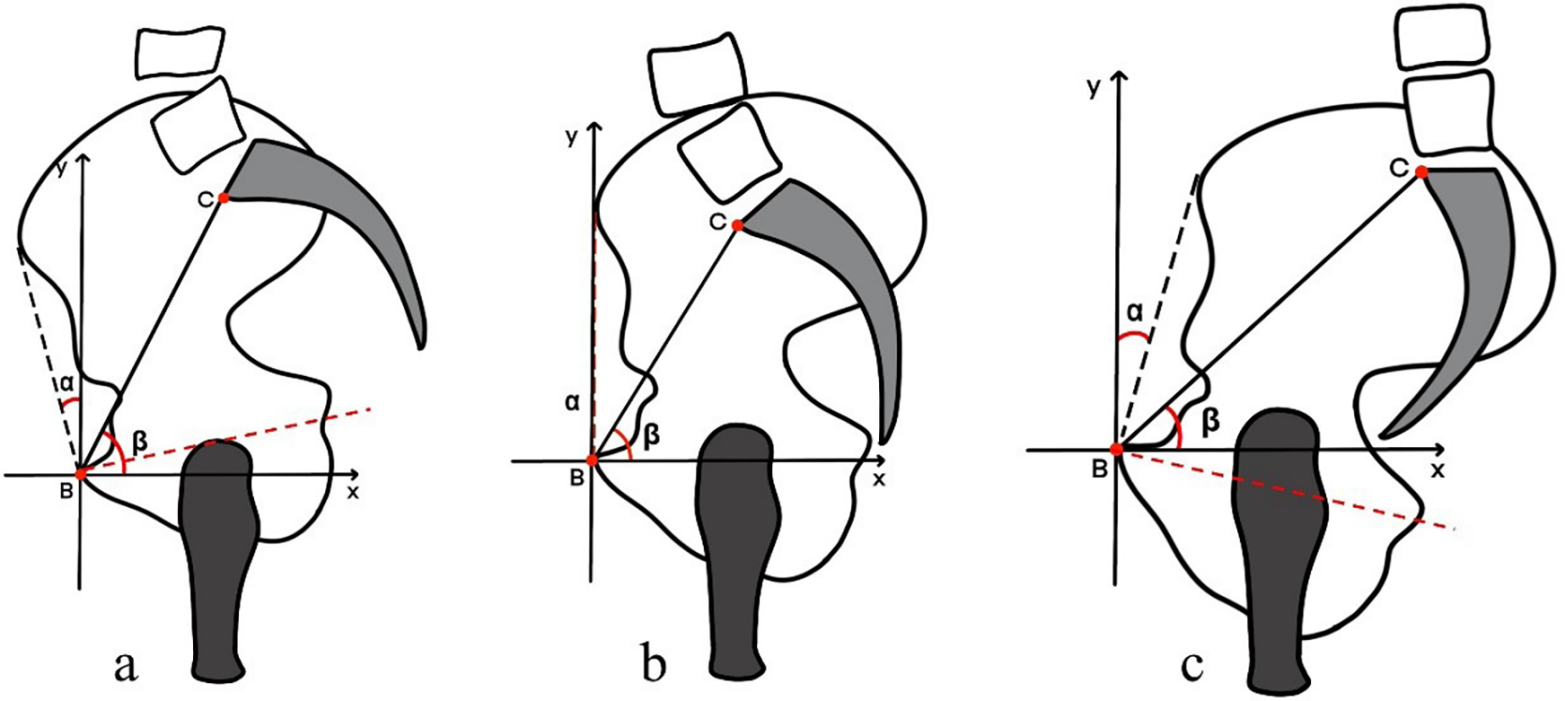
Figure illustrates the three different states of pelvic orientation: Panel a: Shows the pelvis in an anterior tilt position (pelvic anteversion). In this state, the front of the pelvis tilts downward, increasing the lumbar lordosis. Panel b: Depicts the pelvis in a neutral position, where the pelvic orientation is balanced without excessive tilting in either direction. Panel c: Illustrates the pelvis in a posterior tilt position (pelvic retroversion). Here, the pelvis tilts backward, flattening the lower back curve and reducing lumbar lordosis.

Pelvic sacral angle (PSA) is defined as the angle between the line extending from the anterior point of the sacral endplate to the superior margin of the pubic symphysis and the horizontal line. For clarity, this angle is abbreviated as PSA. Anterior pelvis plane angle (APPA) is defined as the angle between the anterior pelvic plane and the line connecting the superior margin of the pubic symphysis to the anterior superior iliac spine. When the anterior pelvic plane (APP) is positioned anterior to the vertical line, the APPA is considered negative. Conversely, if the APP is posterior to the vertical line, the APPA is positive. The angle is recorded as 0° when the APP plane coincides with the vertical line (refers to Figure 2b).

In a lateral x-ray, the iliac cortical density line appears as a straight line several centimeters in length along the anterior border of the S1 vertebra. Iliac tilt (IT) is defined as the angle between the iliac cortical density line and a vertical line. If the iliac cortical density lines on both sides do not coincide, record the angles for each side separately as IT superior (ITs) and IT inferior (ITi).

### Evaluation of intra-rater and inter-rater reliability

Two spine surgeons conducted radiographic measurements utilizing the PACS software. To evaluate intra-rater reliability, each rater measured all radiographic parameters a second time, with an interval of 2–3 weeks following the initial assessment. Throughout the evaluation period for inter-rater reliability, each rater remained unaware of the measurements made by the other. Measurement reliability was assessed using two-way random, single-measure intraclass correlation coefficients (ICC 2,1) with 95% confidence intervals. ICC values were interpreted according to standard guidelines: <0.40 as poor, 0.40–0.59 as fair, 0.60–0.74 as good, and >0.75 as excellent reliability.

## Statistical methods

Data analysis was conducted using SPSS software (version 24; SPSS, Inc., Chicago, IL). The Shapiro–Wilk test assessed the normality of continuous variables, while Chi-square tests were applied to categorical variables. A subgroup analysis by biological sex evaluated male/female differences in parameters. Independent sample T-tests compared differences in PI, PT, SS, PSA, APPA, ITs, ITi, LL, PHD, SFPl,and SFPr between males and females. Paired t-tests analyzed differences between right and left SFP, as well as between superior and inferior IT. Sex differences in Risser sign grading were examined using Chi-square tests. Pearson correlation analysis explored relationships between all parameters, with correlation coefficients interpreted as follows: 0.00–0.19 as very weak, 0.20–0.39 as weak, 0.40–0.59 as moderate, 0.60–.79 as strong, and 0.80–1.00 as very strong correlation. Measurement reliability was evaluated using intraclass correlation coefficients (ICCs). Statistical significance was set at 0.05.

## Results

### Basic information

This study included a total of 354 adolescent participants, with a mean age of 11.41 ± 2.47 years (range 6–18 years). Of these participants, 155 were boys and 199 were girls. At the time of participation, 60 subjects were classified as Risser sign stage 0, 14 were at stage 1, 62 were at stage 2, 96 were at stage 3, 78 were at stage 4, and 44 were at stage 5.

### Comparison of spinopelvic coronal and sagittal parameters between males and females

To examine the differences in spine-pelvic parameters between genders, we conducted a subgroup analysis based on biological sex. The specific results are presented in Table 1. Significant differences were observed in the Risser sign and PT. Among males, the distribution of the Risser sign was as follows: 30 at grade 0, 10 at grade 1, 37 at grade 2, 36 at grade 3, 28 at grade 4, and 14 at grade 5. Among females, the distribution was: 30 at grade 0, 4 at grade 1, 25 at grade 2, 60 at grade 3, 50 at grade 4, and 30 at grade 5. This indicates that females generally exhibit more advanced Risser sign gradations compared to males, suggesting higher skeletal maturity in females. The PT values for males were 6.77 ± 7.15° (95% CI: 5.23–8.31, *p* < 0.01), while for females, they were 8.4 ± 7.77° (95% CI: 6.75–10.05, *p* < 0.01).

**Table 1:** Comparison of spinopelvic parameters between male and female adolescents.

Risser sign	Male	Female	t	*p*
			17.72	<0.01
0	30	30		
1	10	4		
2	37	25		
3	36	60		
4	28	50		
5	14	30		
Age	11.32 ± 2.5	11.48 ± 2.44	−0.586	0.559
PI	42.24 ± 8.57	41.78 ± 8.77	0.500	0.617
PT	6.77 ± 7.15	8.4 ± 7.77	−2.028	0.043*
SS	33.91 ± 8.52	33.9 ± 9.37	0.015	0.988
PSA	67.44 ± 6.94	68.12 ± 8.45	−0.815	0.416
APPA	−4.78 ± 7.74	−5.41 ± 7.38	0.780	0.436
ITs	67.35 ± 7.49	68.33 ± 7.31	−1.235	0.218
ITi	66.86 ± 8.3	67.78 ± 7.25	−1.101	0.272
LL	46.83 ± 12.54	48.18 ± 14.14	−0.936	0.350
PHD	37.55 ± 6.75	38.77 ± 7.56	−1.584	0.114
SFPl	68.74 ± 5.58	68.2 ± 5.11	0.945	0.345
SFPr	66.86 ± 8.3	67.78 ± 7.25	−1.632	0.104

PSA, the pelvic sacral angle; APPA, anterior pelvis plane angle; ITs, IT superior; ITi, IT inferior; PHD, the Pubo-Hilgenreiner Distance; SFPl, the left side of sacral-femoral-pubic angle; SFPr, the right side of sacral-femoral-pubic angle.

* *p* < 0.05 indicates that the difference is statistically significant.

### Relationship between coronal parameters and sagittal parameters

A significant positive correlation exists between the Risser sign and PI (r = 0.21), indicating that PI increases with bone maturity. Similarly, a positive correlation is observed between the Risser sign and PT (r = 0.22). However, the correlations between the Risser sign and other variables are either weaker or not significant.

A moderate positive correlation is noted between PI and PT (r = 0.41), suggesting that both parameters tend to increase together. There is a strong positive correlation between PI and SS (r = 0.57), highlighting a close relationship. Conversely, PI and PSA exhibit a negative correlation (r = −0.43), where an increase in PI corresponds to a decrease in PSA.

A negative correlation exists between PT and SS (r = −0.48), indicating that an increase in PT is associated with a decrease in SS. A very strong negative correlation was observed between PT and PSA (r = −0.89), indicating that as PSA increases, PT decreases, leading to greater pelvic anteversion (refers to Figure 2a). Additionally, a strong positive correlation is found between PT and APPA (r = 0.60), indicating that both PT and APPA increase together. PT shows strong negative correlations with ITs (r = −0.76) and ITi (r = −0.75).

SS and PSA are positively correlated (r = 0.40), suggesting that an increase in SS is associated with an increase in PSA. SS also shows strong positive correlations with ITs (r = 0.56) and ITi (r = 0.57), indicating a close relationship between SS and these parameters. A very strong positive correlation is noted between SS and LL (r = 0.83).

A strong negative correlation exists between PSA and APPA (r = −0.67), indicating that an increase in PSA corresponds to a decrease in APPA. PSA shows very strong positive correlations with ITs (r = 0.81) and ITi (r = 0.81), suggesting a close relationship between PSA and these measures. APPA shows strong negative correlations with ITs (r = −0.61) and ITi (r = −0.60), indicating that as APPA increases, these measures decrease. There is a very strong positive correlation between ITs and ITi (r = 0.99).

LL exhibits a weak positive correlation with PHD (r = 0.19). Moderate positive correlations are observed between LL and SFPl (r = 0.42) and SFPr (r = 0.43). PHD shows moderate positive correlations with SFPl (r = 0.41) and SFPr (r = 0.38). There is a very strong positive correlation between SFPl and SFPr (r = 0.92) (see Table 2 for details).

**Table 2:** Correlation matrix of various spinopelvic parameters.

	Risser	PI	PT	SS	PSA	APPA	ITs	ITi	LL	PHD	SFPl	SFPr
Risser	1											
PI	0.21*	1										
PT	0.22*	0.41*	1									
SS	−0.01	0.57*	−0.48*	1								
PSA	−0.25*	−0.43*	−0.89*	0.40*	1							
APPA	0.04	0.14	0.60	−0.42	−0.67*	1						
ITs	0.17	−0.13	−0.76*	0.56*	0.81*	−0.61*	1					
ITi	0.16	−0.12	−0.75*	0.57*	0.81*	−0.60*	0.99*	1				
LL	−0.17	0.43*	−0.45*	0.83*	0.41*	−0.37*	0.55*	0.56*	1			
PHD	0.42*	0.01	−0.26*	0.19	0.25*	−0.36*	0.31*	0.30*	0.19*	1		
SFPl	−0.23*	−0.13	−0.59*	0.41*	0.63*	−0.57*	0.59*	0.60*	0.42*	0.41*	1	
SFPr	−0.28*	−0.15	−0.60*	0.39*	0.62*	−0.53*	0.52*	0.58*	0.43*	0.38*	0.92*	1

* *p* < 0.05 indicates statistical significance.

The Intraclass Correlation Coefficients (ICCs) for the various parameters indicate uniformly high reliability. These findings collectively demonstrate that the measurements across all parameters are consistently reliable, with ICC values reflecting excellent reliability (see Table 3 for details).

**Table 3:** Reliability assessment of spinopelvic parameter measurements using intraclass correlation coefficients.

Parameter	Intra-rater ICC (95% CI)	Inter-rater ICC (95% CI)
PI	0.94 (0.91–0.96)	0.92 (0.89–0.95)
PT	0.92 (0.89–0.94)	0.90 (0.87–0.93)
SS	0.93 (0.90–0.95)	0.91 (0.88–0.94)
PSA	0.91 (0.88–0.93)	0.89 (0.86–0.92)
APPA	0.95 (0.92–0.97)	0.93 (0.90–0.96)
ITs	0.94 (0.91–0.96)	0.92 (0.89–0.95)
ITi	0.93 (0.90–0.95)	0.91 (0.88–0.94)
LL	0.92 (0.89–0.94)	0.90 (0.87–0.93)
PHD	0.91 (0.88–0.93)	0.89 (0.86–0.92)
SFPl	0.94 (0.91–0.96)	0.92 (0.89–0.95)
SFPr	0.93 (0.90–0.95)	0.91 (0.88–0.94)

## Discussion

Our radiographic analysis of 354 asymptomatic Chinese Han adolescents revealed several significant relationships between pelvic coronal parameters and sagittal balance measures. Key findings include: (1) significant gender differences in PT and Risser sign distribution, with females exhibiting more advanced skeletal maturity; (2) moderate to strong correlations between coronal parameters (SFP angle, PHD) and pelvic sagittal parameters (PT, SS, PSA); and (3) high correlation between newly introduced parameters (IT, PSA) and established sagittal plane measurements. The strong negative correlation we observed between PT and PSA (r = −0.89) indicates that these parameters offer complementary information about pelvic orientation. As PSA increases, PT decreases, leading to greater pelvic anteversion (Figure 2a). This relationship could provide surgeons with alternative methods to assess pelvic sagittal balance, particularly when traditional measurements may be challenging to obtain accurately.

AIS presents as a three-dimensional deformity with significant impact on sagittal alignment, where the pelvis plays a compensatory role through anterior and posterior tilting mechanisms to maintain global balance. This pelvic orientation serves as a critical patient-specific parameter in both spine and hip surgical planning, with its sagittal positioning increasingly recognized as fundamental to optimizing outcomes in pediatric spinal surgery (17–21). PI is a key morphological parameter that remains constant in adults but may change during growth. Recent research has also shown that changes in PI are associated with sacroiliac joint function (22). PT is clinically significant for evaluating the global sagittal alignment in patients with spinal deformities (23). During the compensation of kyphosis, changes in pelvic position are notable, and PT serves as a reliable indicator for detecting these changes. Recently, PT has been utilized as a reference in surgical planning (24). A study involving 626 asymptomatic individuals found significant gender-specific differences in sagittal spinal alignment (25). Females generally exhibited greater LL and PT compared to males. These differences are attributed to anatomical and hormonal variations between genders, affecting spinal biomechanics and alignment​. Therefore, this study aims to include asymptomatic adolescents to analyze their spinopelvic parameters, examining the correlation between pelvic coronal parameters and sagittal balance parameters to validate the use of pelvic coronal parameters as preliminary or supplementary indicators in assessing pelvic rotation.

In this study, we observed in pediatric spines, PT is often minimal, with some subjects even exhibiting negative PT values (Refer to Figure 2a, which illustrates pelvic anterversion), which is inconsistent with the posterior compensatory mechanisms seen in degenerative spines (seen in Figure 2c) (26). Describing the sagittal plane of the pelvis solely by PT is insufficient. Previous studies have investigated the impact of pelvic rotation (rotation of the pelvis around the craniocaudal axis) and pelvic tilt (rotation of the pelvis around the left-right axis, sometimes referred to as flexion/extension or inclination) on measurements performed on pelvic radiographs (27). Various methods have been proposed to define these parameters. Among them, the PSA and APPA are accepted indicators in spine and joint surgery, providing valuable references for pelvic spatial positioning (28, 29). Liu C et al. (30) defined the anterior pelvic plane as the plane crossing the two anterosuperior iliac spines (ASISs) and the pubic tubercles, representing the anatomical PT from its sagittal projection. Thoms et al. (31) defined the anteroposterior pelvic inlet as the line connecting the superior pubic symphysis and the sacral promontory. These angles quantify a similar concept of pelvic orientation. Therefore, in the present study, we measured the APPA and PSA with respect to PT. APPA is approximately 4° when normal adult subjects are in their natural standing position in previous studies (32). In our study, the APPA data for minors was −5.14 ± 7.54°, ranging from −26.78 to 16.92°. No gender differences were observed, indicating that minors have a wider range and lower mean value of APPA.

IT was first reported by Doi (9), who suggested that it can be easily and directly measured using the iliac cortical density line, providing a reliable estimation of the PT. Our results also indicate a strong association between IT and PT, supporting its clinical utility. Interestingly, we observed no significant difference in ITs formed by the upper and lower iliac cortical density lines (see Table 4), with frequent overlapping of these lines. This alignment pattern may indicate balanced biomechanical forces acting on both sides of the pelvis in asymptomatic adolescents, potentially serving as a reference standard for normal pelvic symmetry. Deviations from this pattern in patients with AIS might signify compensatory pelvic mechanisms or structural asymmetries that could influence treatment planning. Additionally, the density line serves as an anatomical reference for segmenting the pelvis, offering a natural delineation line given the irregular structure of the pelvis. Future research should investigate whether asymmetry of these lines correlates with curve progression or compensatory mechanisms in scoliotic populations.

**Table 4:** Comparative analysis of left vs. right sacro-femoral-pubic angle and superior vs. inferior iliac tilt.

Comparison	t	*p*
SFP left vs. right	0.460	0.646
ITs vs. ITi	0.920	0.358

The precise correlation between specific morphological changes in spinopelvic parameters, as identified on AP pelvic radiographs or lateral lumbar spine radiographs, and their impact on spinopelvic balance assessed in the sagittal plane remains inadequately understood (33). The pelvic plain radiograph is a two-dimensional projection of a three-dimensional structure, inherently containing information about the sagittal plane (34). Thus, it is necessary to incorporate frontal parameters when assessing the sagittal state of the pelvis.

**Figure 3:**
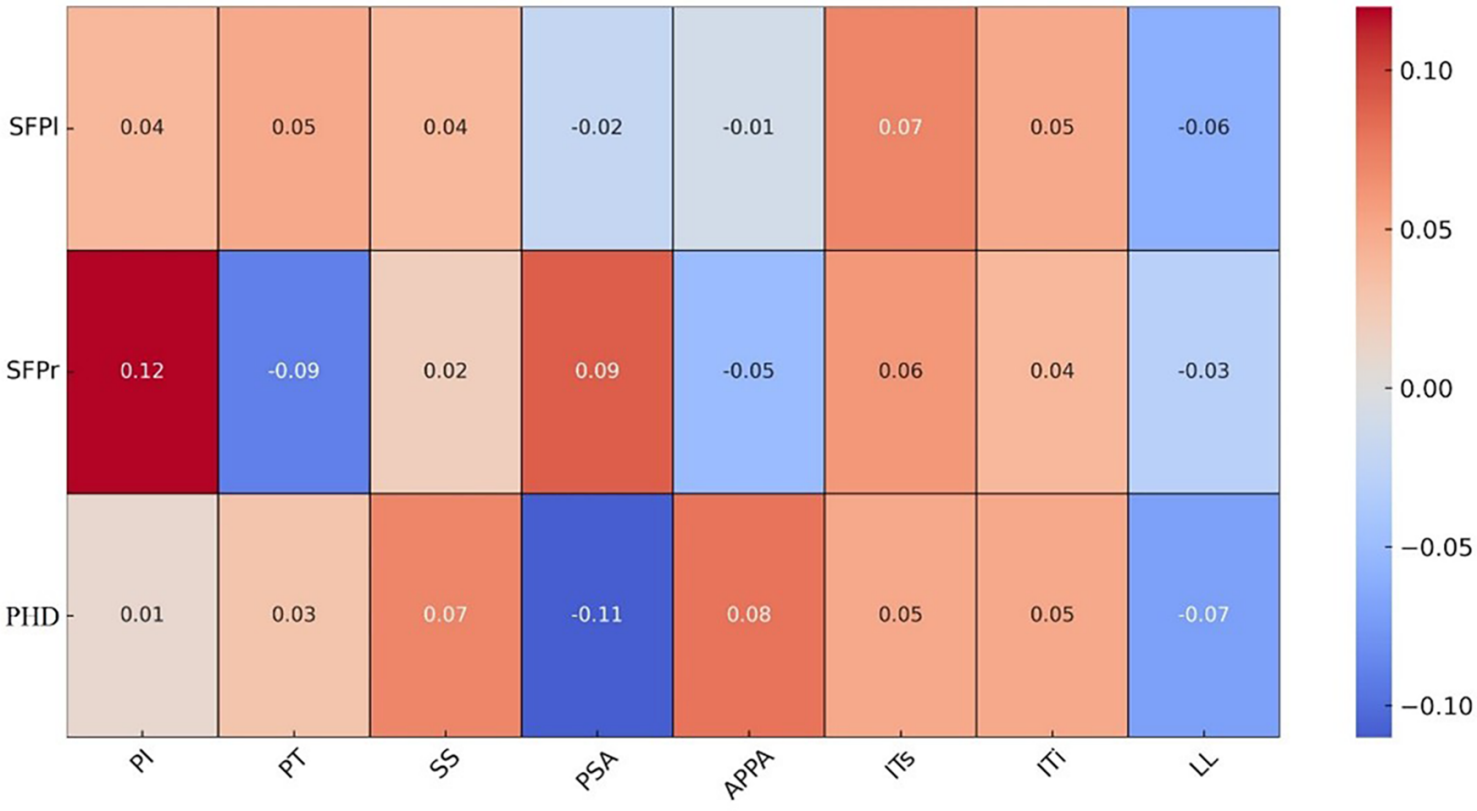
Figure presents an analysis of the correlation between coronal and sagittal plane parameters of the pelvis.

In this study, the measurements of SFP on both the left and right sides were consistent, aligning with previous literature (7 35–36). Our findings align with previous research by Blondel B. et al. demonstrating the utility of SFP as a coronal parameter. However, further studies are needed to confirm these findings in larger cohorts. Our analysis demonstrated strong correlations between SFP and PT, SS, PSA, APPA, ITs, and ITi, indicating a close relationship between SFP and the sagittal plane status of the pelvis (seen in Figure 3). Significant correlations were also observed between SFP and both IT and PHD, suggesting that both distance and angular parameters of the frontal pelvis are related to the sagittal plane. Based on the correlation results, we consider that larger SFP and PHD values indicate a more anteriorly tilted pelvis in the sagittal plane, with larger PSA and SS values and smaller PT and APPA values. A larger SFP angle is often associated with a longer PHD distance.

The Pubo-Hilgenreiner Distance (PHD) is a new parameter defined as the distance between the upper border of the pubic symphysis and the line connecting the outer edges of the bilateral acetabula. This measurement was inspired by the evaluation of hip development (37). As the triradiate cartilage closes, measuring Hilgenreiner's line becomes less reliable. Therefore, we adopted the outer edge of the acetabulum as a bony landmark, which can be clearly identified in both children and older minors (see Figure 1). PHD is also unaffected by gender and shows significant correlations with several parameters in this study. For instance, it has positive correlations with the Risser sign, PI, IT, and PSA, and a significant negative correlation with PT and APPA. Our findings demonstrate clear clinical advantages of incorporating coronal parameters when assessing sagittal balance. First, coronal radiographs are more routinely obtained in clinical practice than sagittal views, making parameters like SFP angle and PHD particularly valuable when complete imaging is unavailable. Second, these coronal measurements offer supplementary information that can help confirm sagittal balance assessments. Third, the strong correlations between coronal parameters (SFP, PHD) and sagittal measures (PT, PSA) suggest that these measurements could potentially be used to develop predictive models that estimate sagittal alignment from coronal radiographs alone, reducing radiation exposure by potentially eliminating the need for additional imaging. Finally, in pediatric patients where minimizing radiation is particularly important, the ability to extract sagittal balance information from necessary coronal radiographs represents a significant clinical advantage. While we do not suggest that coronal parameters should replace established sagittal measurements, our data supports their use as valuable complementary tools that can enhance clinical decision-making in the evaluation and management of adolescent spinal conditions.

The growth rates between boys and girls are not uniform. Table 1 in this study demonstrates significant gender differences in the distribution of the Risser sign. Previous research has analyzed spinal balance parameters in adolescents (38), and our earlier studies have also demonstrated differences in PT between genders. Additionally, the differences in PT among various age groups were found to be significant (5, 39). This indicates that the state of PT is related not only to gender but also to age, which may be associated with anatomical differences in the pelvis and varying compensatory mechanisms.

### Limitations

This study has several limitations. First, as a cross-sectional study, we cannot determine causality between the assessed parameters; thus, only associations can be concluded. Since this study exclusively included healthy subjects, the relationships between parameters in a normal population were preliminarily explored. Future research will extend this analysis to include individuals with scoliosis. Second, the measurements in this study were not based on CT three-dimensional reconstructions of the pelvis, making it unavoidable to have inaccuracies due to pelvic rotation. Third, the pelvis exhibits three-dimensional motion, involving combined movements in different directions. Our study only measured and observed uniplanar motion without considering the impact of multidirectional movements. Further investigations with larger sample sizes are necessary to confirm these findings.

## Conclusion

Based on our results, we believe that parameters such as IT, PSA, SPF, and PHD are correlated with traditional spinopelvic parameters and can be clinically used to assess pelvic sagittal balance. Future research should focus on longitudinal studies to determine the predictive value of coronal parameters in the progression of AIS. The state of the pelvis in minors should be identified and adequately considered in the evaluation and treatment plans for AIS.

## Data Availability

The original data presented in the study are included in the article/Supplementary Material, further inquiries can be directed to the corresponding author.
